# Endoscopic view of the vertebra during laser balloon ablation of paroxysmal atrial fibrillation

**DOI:** 10.1002/ccr3.8463

**Published:** 2024-02-01

**Authors:** Shun Sasaki, Yuki Shibuya, Hitoshi Minamiguchi, Takashige Sakio, Yuma Hamanaka, Takashi Kanda, Yasuhiro Ichibori, Nobuhiko Makino, Atsushi Hirayama, Yoshiharu Higuchi

**Affiliations:** ^1^ Cardiovascular Division Osaka Police Hospital Osaka Japan

**Keywords:** atrial fibrillation, laser balloon, pulmonary vein isolation, vertebra

## Abstract

**Key Clinical Message:**

Left atrial posterior wall on the vertebra is often difficult to obtain stable tissue contact with ablation‐catheter. Laser balloon ablation is effective because the compression from the vertebra can be visualized through endoscopy.

**Abstract:**

When performing pulmonary vein isolation (PVI) with radiofrequency, left atrial posterior wall on the vertebra is often difficult to obtain stable tissue contact with ablation‐catheter because of the movement of the ablation point. Laser balloon ablation is effective for the achievement of durable PVI in cases with such anatomical characteristics because the compression from the vertebra can be visualized through endoscopy.

## 1 CASE IMAGES

**FIGURE 1:**
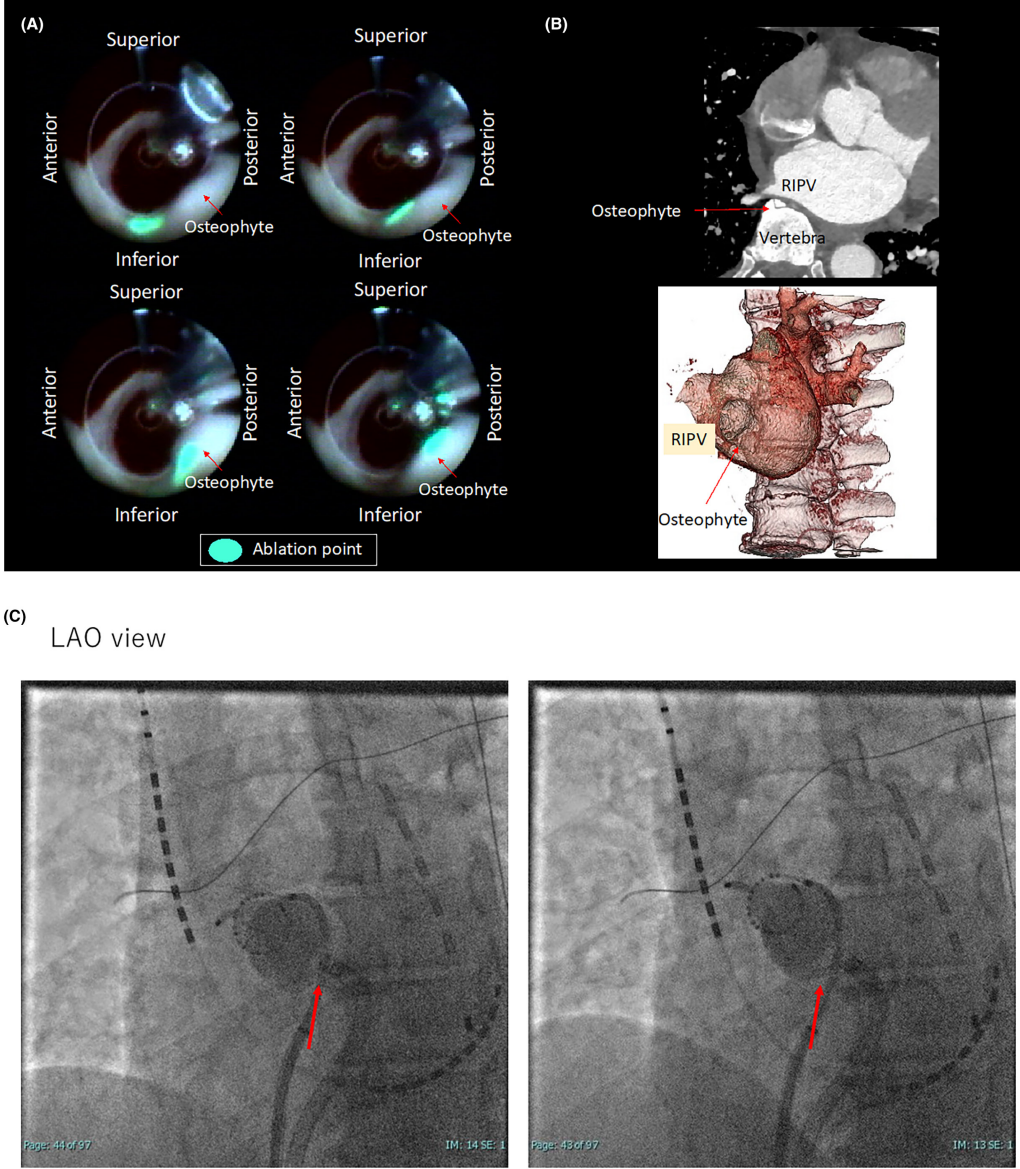
(A) Endoscopic view of the vertebra compressing the left atrial posterior wall. (B) Enhanced computed tomography (axial, 3D) showed the osteophytes of the vertebra pressing against the left atrial posterior wall. (C) Fluoroscopy in left anterior oblique (LAO) view showed the osteophytes of the vertebra pressing against the left atrial posterior wall during respiratory movements during the right inferior pulmonary vein ablation. LAO, left anterior oblique; RIPV, right inferior pulmonary vein.

**FIGURE 2:**
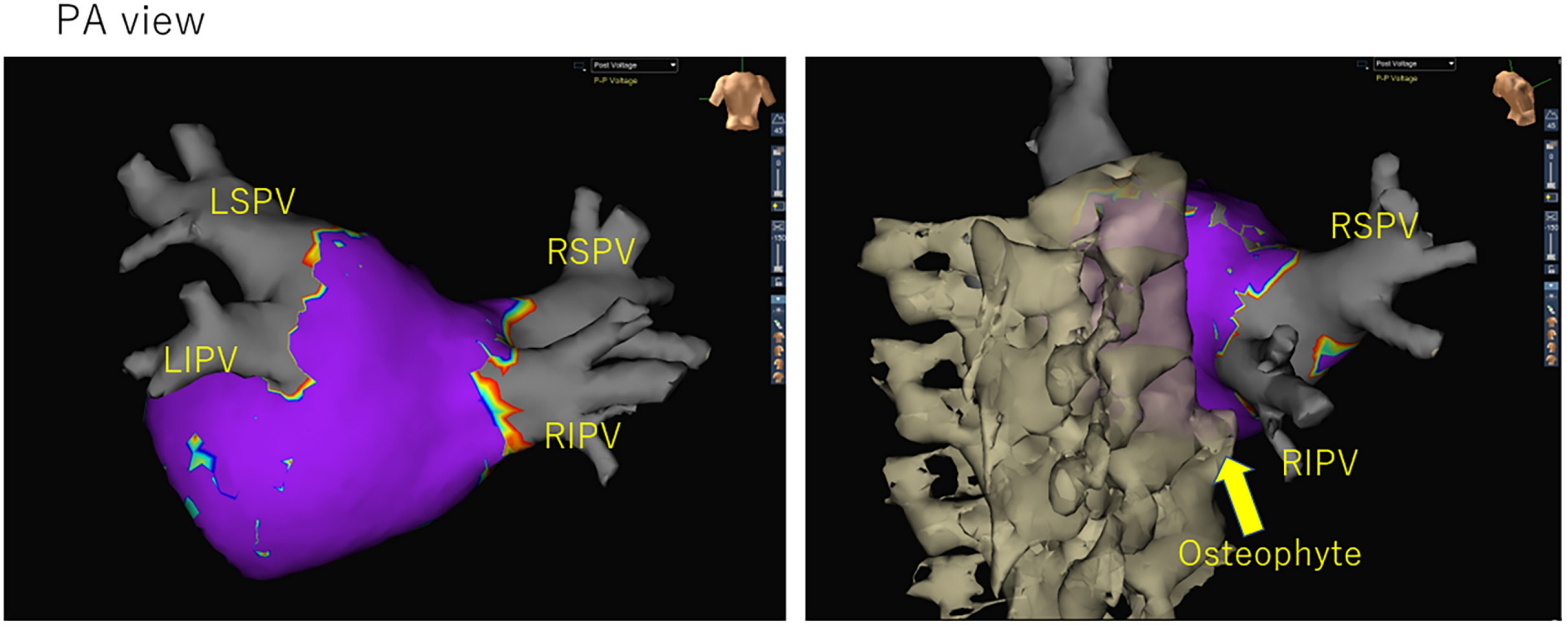
After pulmonary vein (PV) isolation, left atrial voltage was obtained using a 20‐pole circular catheter with 1 mm electrode (EP star Libero, Japan Lifeline Co., Tokyo, Japan) with three‐dimensional electroanatomical mapping (NavX System, St. Jude Medical Inc., St. Paul, MN, USA). Purple showed for healthy tissue (electrograms > 0.5 mV) and gray for scar tissue (electrograms < 0.2 mV). All PVs were successfully isolated. LIPV, left inferior pulmonary vein; LPSV, left superior pulmonary vein; PA, posteroanterior; RIPV, right inferior pulmonary vein; RSPV, right superior pulmonary vein.

A 66‐year‐old male was admitted for pulmonary vein isolation (PVI) using first‐generation laser balloon (HeartLight, CardioFocus, Marlborough, MA, USA) for paroxysmal atrial fibrillation. Echocardiography showed that the left atrial diameter was 51 mm and there was no valvular disease. Contrast cardiac computed tomography (CT) showed four pulmonary veins (PVs) were all present individually and no common ducts were observed. Laser energy was used point‐by‐point, overlapping each lesion by 30%–50%. The energy level was targeted to 8.5 W or more, while the anterior wall of the PVs was treated with 12 W.^1^ When isolating the right inferior PV, the left atrial posterior wall, which was compressed by the vertebra, was visualized through the endoscope of the laser balloon (Figure 1). CT scan and fluoroscopy also showed osteophytes of the vertebra pressing against the left atrial posterior wall in the same area (Figure 1B,C). Visualization of the lesion enabled the achievement of PVI without any gaps on the isolation line even though the ablation point was moving dynamically (Figure 1A). Finally, all PVs were successfully isolated (Figure 2).

When performing the PVI with radiofrequency, the left atrial posterior wall on the vertebra is often difficult to obtain good tissue contact with the ablation‐catheter because of the movement of the ablation point. However, the visualization of its movement has been rarely reported. Direct visualization of the ablation points and its lesion is unique advantage of the visually guided laser balloon. Laser balloon ablation is presumably effective for the achievement of a durable PVI in cases with such anatomical characteristics.

## AUTHOR CONTRIBUTIONS

**Shun Sasaki:** Data curation; writing – original draft. **Yuki Shibuya:** Investigation. **Hitoshi Minamiguchi:** Conceptualization; data curation; project administration; writing – original draft; writing – review and editing. **Takashige Sakio:** Data curation. **Yuma Hamanaka:** Investigation. **Takashi Kanda:** Investigation. **Yasuhiro Ichibori:** Investigation. **Nobuhiko Makino:** Data curation; investigation; project administration. **Atsushi Hirayama:** Project administration. **Yoshiharu Higuchi:** Project administration.

## FUNDING INFORMATION

None.

## CONFLICT OF INTEREST STATEMENT

The authors declare no conflicts of interest.

## CONSENT

Written informed consent was obtained from the patient to publish this report in accordance with the journal's patient consent policy.

## Data Availability

The data that support the findings of this study are available from the corresponding author upon reasonable request.
